# B-Cell Acute Lymphoblastic Leukaemia of the Uterus

**DOI:** 10.5334/jbsr.2211

**Published:** 2020-11-09

**Authors:** Eva Vanheule, Rudy Van den Broecke, Pieter De Visschere

**Affiliations:** 1 Ghent University, BE

**Keywords:** Acute lymphoblastic leukaemia, uterus, female reproductive tract, lymphoma, urogenital MRI

## Abstract

**Teaching Point:** Uterine involvement of B-cell acute lymphoblastic leukaemia is exceedingly rare. Globular enlargement of the uterus with diffuse homogeneous signal intensity is suggestive for the diagnosis on magnetic resonance imaging (MRI) in clinically suspected cases.

## Case History

A 41-year-old woman presented at the emergency department with severe metrorrhagia. It had started a few weeks ago and was gradually increasing in severity. A blood transfusion was even needed to compensate for the anaemia. A hemorrhagic myoma uteri was initially suspected on clinical examination but upon further inquiries, the patient stated she was recently diagnosed with relapsed B-cell acute lymphoblastic leukaemia (B-ALL).

**Figure 1:**
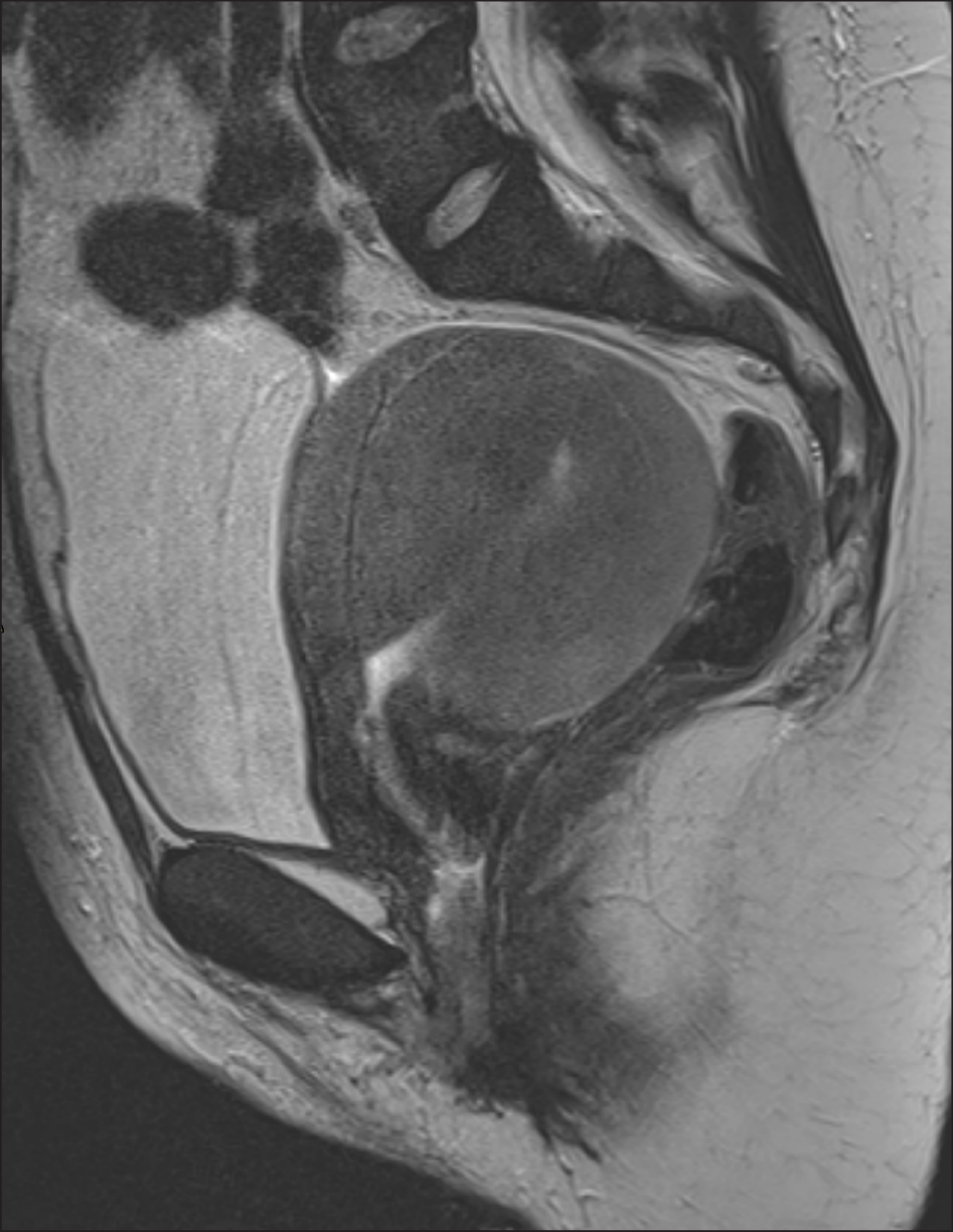


**Figure 2:**
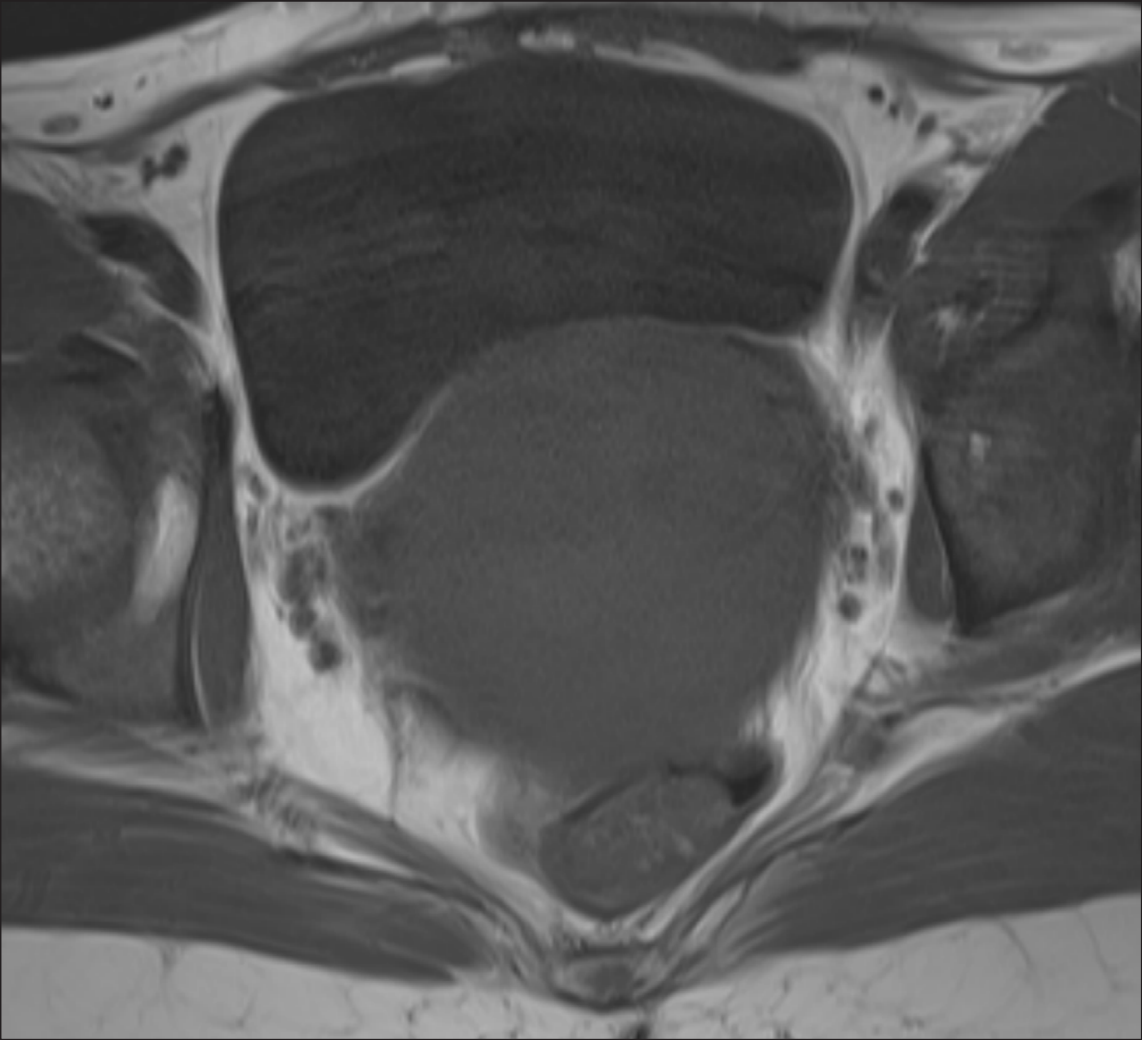


**Figure 3:**
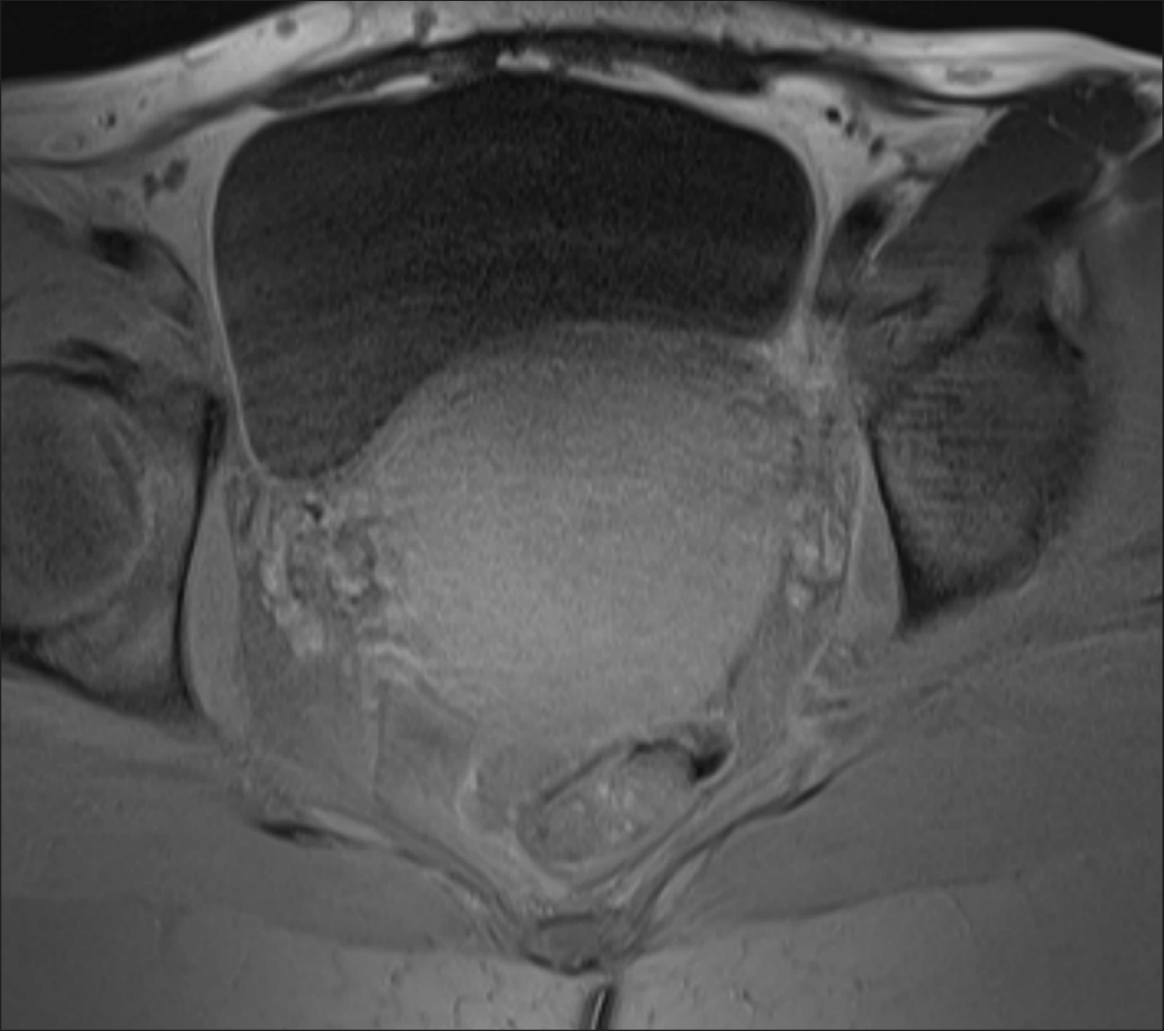


As a first imaging evaluation ultrasound was performed, showing a non-specific uterine enlargement. Magnetic resonance imaging (MRI) of the pelvis was added for further exploration. MRI confirmed the diffuse uterine enlargement and demonstrated a thickened, poorly defined (nearly ‘washed out’) junctional zone. The uterine signal was homogeneously slightly hyperintense on T2-weighted images (Figure 1) and homogeneous hypo- to isointense on T1-weighted images, with slight decrease after fat suppression (Figures 2 and 3). Parametric structures were normal. Enlarged para-iliacal and retroperitoneal lymph nodes were noted (not shown). The clinical and imaging findings were suggestive of haematological invasion of the uterus by the B-ALL rather than a hemorrhagic myoma. A biopsy was taken from the uterus and the pathology result confirmed the diagnosis of B-ALL of the uterus.

## Comment

B-ALL is an acute malignancy of immature lymphocytes in the bone marrow and blood. The most commonly affected extramedullary sites are the testes and the central nervous system. Infiltration of the female reproductive system is often found microscopically in relapsed B-ALL, but it is rarely clinically obvious. Furthermore, the uterus is rarely the dominant site of involvement [1]. The clinical symptoms of uterine involvement are nonspecific such as vaginal bleeding, discharge or abdominal pain, making it difficult to consider the diagnosis based on clinical examination [1].

Ultrasound is the first choice for imaging investigation, and although it can provide an initial evaluation of uterine mass, MRI is recommended for further assessment. Global enlargement of the uterus due to diffuse infiltration of the B-ALL with a homogeneous hypo- to isointense signal on T1-weighted images and slight hyperintense signal on T2-weighted images are most commonly seen. The junctional zone and uterine border are usually spared and pelvic lymphadenopathy is common. This imaging appearance is, however, not specific and the disease can also manifest as multiple nodules or a large lobulated uterine mass. In the latter case, the absence of necrosis despite the large size of the tumour and the intact junctional zone are diagnostic clues for haematological invasion. In rare cases, the architecture may be disturbed and invasion into nearby organs can occur [1]. Treatment of uterine involvement of B-ALL is chemotherapy but the prognosis is poor.
